# Heart Rate Variability Alterations During Delayed-Onset Muscle Soreness-Inducing Exercise—With Piezo2 Interpretation

**DOI:** 10.3390/sports13080262

**Published:** 2025-08-10

**Authors:** Gergely Langmár, Tekla Sümegi, Benjámin Fülöp, Lilla Pozsgai, Tamás Mocsai, Miklós Tóth, Levente Rácz, Bence Kopper, András Dér, András Búzás, Balázs Sonkodi

**Affiliations:** 1 Faculty of Kinesiology, Hungarian University of Sports Science, 1124 Budapest, Hungary; 2 Hungarian National Academy of Handball, 8630 Balatonboglár, Hungary; 3 Department of Social Sciences, Faculty of Health Sciences, Semmelweis University, 1085 Budapest, Hungary; 4 Department of Health Sciences and Sport Medicine, Hungarian University of Sports Science, 1123 Budapest, Hungary; 5 Faculty of Health Sciences, Institute of Physiotherapy and Sport Science, University of Pécs, 7622 Pécs, Hungary; 6 Physical Activity Research Group, Szentágothai Research Center, University of Pécs, 7622 Pécs, Hungary; 7 Institute of Laboratory Medicine, Semmelweis University, 1085 Budapest, Hungary; 8 Institute of Biophysics, HUN-REN Biological Research Center, 6726 Szeged, Hungary; 9 Department of Sport Medicine, Semmelweis University, 1122 Budapest, Hungary

**Keywords:** sport injury risk, entropy, oxidative stress, nonlinear parameters, Piezo2 channelopathy, proton

## Abstract

Heart rate variability (HRV) is often modulated by pain; therefore, the objective of this study was to assess whether the induction of delayed-onset muscle soreness (DOMS) is already affected by HRV alterations during exercise, in spite of the fact that pain evolves only post-exercise. An isokinetic dynamometer was used to induce DOMS in this study on 19 young male elite handball players who were subjected to HRV measurements throughout a DOMS-inducing exercise session. The result of this study indicated that the heart rate (HR) dependence of time–frequency domain parameters could be described by an exponential-like function, while entropy showed a V-shaped function, with a minimum “turning point” separated by descending and ascending intervals. The DOMS protocol upshifted the time–frequency domain HRV parameters in the entire HR range, contrary to the sample entropy values that were systematically downshifted, indicative of an upregulated sympathetic tone. The group-averaged HR-dependent sample entropy function showed a nonlinear character under exercise, with lower values for higher DOMS than for the group with lower DOMS below the turning-point HR, and vice versa above it. The differences between the respective HRV(HR) point sets representing the low-DOMS and high-DOMS groups were quantified using a statistical method and found to be significant at the current sample size for all the HRV parameters used. Since oxidative stress is implicated in DOMS, we are the first to report that nonlinear alterations may impact HRV in a HR-dependent manner in DOMS using a Piezo2 interpretation. This finding provides further indirect evidence for an initiating neural microdamage that prevails under DOMS-inducing exercise, and the diagnostic detection of this point may provide control for avoiding further injury risk in sports and exercise activities.

## 1. Introduction

Delayed-onset muscle soreness (DOMS) is an enigmatic pain condition affecting nearly everyone. No unequivocal mechanism for its cause has been discovered since the American physician Theodore Hough first described it more than 125 years ago [1]. The most circulated theories implicate lactic acid, muscle spasm, inflammation, connective tissue damage, muscle damage, and enzyme efflux as the key mechanisms causing DOMS [2]. It is known that unaccustomed and/or strenuous eccentric and/or isometric contractions can induce DOMS. The following symptoms are known to be the consequence of DOMS: muscle stiffness, swelling, loss of force-generating capacity, reduced joint range of motion, and, even more importantly, delayed onset of pain sensation and impaired proprioception [3]. The transient pain sensation develops at about 8 h, peaks at around 1 or 2 days, and subsides within 7 days after the completion of the DOMS-inducing exercise [4]. Thus, DOMS is clearly distinguished from pain that is experienced during or right after exercise [5]. It is noteworthy that heart rate variability (HRV) is impacted by nociceptive C-fibers and pain [6], but, in the case of DOMS, pain is not present during the inducing exercise session and develops only hours later [4].

HRV measures the variation in interbeat intervals from one heartbeat to the next, reflecting changes in the heart rate (HR) over time. Heart rhythm is primarily governed by the synchronized firing of pacemaker cells in the sinoatrial node (SAN) of the heart muscle, which initiates the cardiac cycle. The activity of these pacemaker cells, which primarily generate the so-called orderly “funny” currents via hyperpolarization-activated cyclic nucleotide-gated (HCN) channels, is regulated by the autonomic nervous system (ANS). The complex actions of the underlying interdependent regulatory mechanisms give rise to the variability in the length of the cardiac cycle over different time scales, supporting the optimal performance of the heart under homeostasis. HRV measurement is one diagnostic tool used to detect ANS changes, and it is often used in both recreational and competitive sport activities. Moreover, this technique is proven to be effective for following the proper course of regeneration after strenuous exercise activities. Accordingly, the return of the parasympathetic drive to pre-exercise level, and to even higher magnitudes, is of interest on the path to regeneration. Anaerobic exercise substantially delays the return of parasympathetic tone, or autonomic control, compared to aerobic exercise [7], while strenuous exercise delays this return by two days [8], which is suggested to overlap with the time window for Piezo2 channelopathy [9]. Higher HRV is often associated with better fitness and heart adaptability, while reduced HRV is commonly linked to various pathological conditions, including congestive heart failure, diabetic neuropathy, mental disorders, and cancer. As has been revealed in previous publications regarding the analysis of HRV data per se, the time–frequency domain, as well as nonlinear HRV parameters, prominently entropy, show the same phenomenon from different viewpoints; hence, they are strongly interrelated, but they still carry independent information [10].

The data reflecting how the ANS exerts its modulation during exercise are accumulating. At low intensities, the sympathetic loading increases, while the parasympathetic loading decreases. Under prolonged sympathetic loading, this parasympathetic tone declines to a point where it is almost entirely withdrawn. Thereafter, the sympathetic drive sustains its activity, although it decreases at higher intensities. The current authors hypothesize that a line of demarcation exists where the ANS regulation of exercise activities flips into a disordered state, or a transient point of no return, reflecting initiating microdamage during exercise, leading to DOMS. It is important to note that those experiencing DOMS are prone to higher injury risk, according to the prevailing view of the scientific community [11]. Certain sports and sports modalities, like eccentric/isometric exercise, are highly affected by DOMS. It has long been known that handball is one of the sports that has a high injury rate, especially among adolescent players [12], including non-contact injuries, such as DOMS [9]. This is why the current study was conducted on young elite handball players. Therefore, if the aforementioned transient point of no return could be detected by HRV measurements, then HRV monitoring devices would gain new diagnostic relevance, serving coaches, athletes, and many others. Moreover, the detection of the suspected transient point of no return would help coaches and athletes to change sports modalities from eccentric/isometric exercise to concentric types in order to avoid greater injury risk.

In support of our theory, accumulating evidence suggests that DOMS begins even during the unaccustomed and/or strenuous exercise activity [11,13,14,15] and not 8 h after, when pain arises. HRV alterations are thought to be modulated by nociceptive stimulation; however, in DOMS, nociceptive nerve fibers only reflect pain hours after the DOMS-inducing exercise [4]. Indeed, a recent pilot study showed orthostatic imbalance right after DOMS-inducing exercise, detected by an orthostatic stress test, which is indicative of ANS dysregulation [14]. This finding not only highlighted that ANS disbalance precedes pain evolvement [14] but also increased our suspicion that a transient point of no return should exist already during DOMS-inducing exercise. However, it is important to note that, to our knowledge, no such “primary damage” point has been detected by an HRV monitoring device so far.

A new neurocentric theory of DOMS hypothesizes that DOMS is a bi-phasic, bi-compartmental, non-contact injury mechanism where the primary damage occurs on Type Ia proprioceptive fiber terminals within the muscle spindle under an acute stress response (ASR) time window [16]. This primary damage is theorized to come in the form of an acquired Piezo2 channelopathy [9]. It is noteworthy that this bi-phasic injury mechanism in relation to DOMS was introduced earlier, but only in extrafusal muscle territories [17,18]. However, DOMS could be induced without muscle damage and only at high exercise intensities when muscle damage may evolve [19,20]. Moreover, this DOMS theory not only incorporated the importance of the line of demarcation between good stress and bad stress, reflected in remodeling and the breach of remodeling, respectively [9], but it also highlighted the role of oxidative stress in the DOMS mechanism [16,21]. Furthermore, oxidative stress is shown to be one of the most significant influencing factors of HRV by its time–frequency domains, but not by nonlinear parameters [22]. Interestingly, the conditions where Piezo2 channelopathy is suspected as the primary damage (the one single initiating cause of aging), e.g., in DOMS, are always associated with autonomic disbalance [9] and oxidative stress [23]. Accordingly, the current authors followed indirect neurocentric tracing by using HRV measurement tools. Moreover, this initiating microdamaging event is proposed to reflect quantum mechanical properties, initiated by proton affinity switch on Piezo2 function, which impairs a novel ultrafast proton-based, long-distance proprioceptive signaling in the nervous system [9,24]. Indeed, Piezo2 is the principal mechanotransductory ion channel responsible for proprioception, as was shown by the team of Nobel laureate Ardem Patapoutian [25]. In fact, DOMS occurs with impaired proprioception [3,13]. In support of this, recent combined electromagnetic stimulation (also called paired-associative electromagnetic stimulation), including both transcranial and peripheral, had a positive therapeutic impact on HRV parameters after DOMS-inducing exercise [26] and on muscle activation, force, and performance measurements [27], as detected by HRV analysis and surface electromyography, respectively. Additional confirmation of the Piezo2 channelopathy involved neurocentric DOMS theory [9], and the aforementioned therapeutic paired-associative electromagnetic stimulation DOMS studies [26,27] showed that Piezo2 is the underlying mediator of magnetic stimulation [28], as was theorized earlier [9]. Hence, a deeper understanding of Piezo2 function would not only help us in the treatment of DOMS but perhaps in its prevention as well.

Low-intensity exercise does not appear to alter low-frequency (LF) power of HRV, in contrast to medium- to high-intensity exercise, which decreases LF power to close to zero values as the sympathetic load increases [29]. Furthermore, a recent study revealed that time–frequency domain HRV parameters are interrelated by nonlinear indices, notably with sample entropy (SampEn), which is a very sensitive measure of ANS control [10]. An earlier paper proposed that the LF power of HRV reflects the activity level of Piezo2 in the baroreceptor of the circulation and the heart [30]. Furthermore, a recent paper suggested that Piezo2 is an ultradian sensor and the only ion channel capable of initiating the theorized novel unaccounted ultrafast proton-based, long-distance proprioceptive signaling in the nervous system [9]. Consequently, Piezo2 may initiate and modulate the ultradian rhythm [31]. Furthermore, the ANS controls ultradian fluctuations through baroreflex sensitivity, and, even more importantly, it is HR-dependent [32]. In support of this, conditional PIEZO2 and PIEZO1 gene knock-out mice not only experience baroreflex failure but also essentially lose blood pressure and heart rate control [33]. Correspondingly, the current authors assign Piezo2 to ultradian control and Piezo1 to diurnal control of blood pressure and HR regulation. Accordingly, Piezo1 indeed participates in the diurnal regulation of certain homeostatic processes [34]. It has been theorized that the ultradian clock is present at the hippocampal end of the novel ultrafast ultradian signaling, arising from the rhythmic bacteria-induced Piezo2-containing enterochromaffin cells–neural complex, as the backbone of the microbiome–gut–brain axis [31]. What is more, this ultradian rhythm, with the contribution of Piezo2 [23], is likely consolidated in learning and memory through the proposed ultradian hippocampal clock in synchrony with rapid eye movement (REM) sleep [31]. In favor, the association between the ultradian rhythm and REM sleep has long been observed [35], and this communication is suggested to involve the temporal pattern of the basic rest–activity cycle [36].

The goal of our study was to detect alterations in HRV measurements during DOMS-inducing exercise sessions that would reflect the suspected initiating neural primary damage, or the transient point of no return in the DOMS onset mechanism, despite pain evolving hours after the inducing exercise.

## 2. Materials and Methods

### 2.1. Participants

In this study, the sample included 19 adolescent male U16 elite handball players. At the time of the data collection procedure, all the participating individuals were players in the Hungarian National Academy of Handball (Balatonboglar, Hungary). Basic anthropometric data of the sample included the following: age: 15.65 ± 0.65 yrs, body mass: 73.68 ± 11.14 kg (Inbody 270, InBody Co., Ltd., Seoul, Republic of Korea), and height: 183.53 ± 6.62 cm (SECA 213, Seca GmbH & Co. KG, Hamburg, Germany). The only players who were included in the sample did not have lower limb pain at the time of data collection or any kind of lower limb injury prior to the measurements. Moreover, we excluded instances where a player’s signal was not accurate enough during the dynamometer test or where the electrode fell off the player’s body. Prior to the measurements, the players were informed about the data collection protocol and the possible risks of this study. Due to the players’ young age, every individual’s parent signed a written consent about the player’s participation in this study according to the Declaration of Helsinki Ethical Research Principles. This study was approved by the Science Ethics Committee of the Hungarian University of Sports Science (Ethical Approval Number: TE-KEB/18/2022).

### 2.2. Measurement Procedure

**Figure 1:**
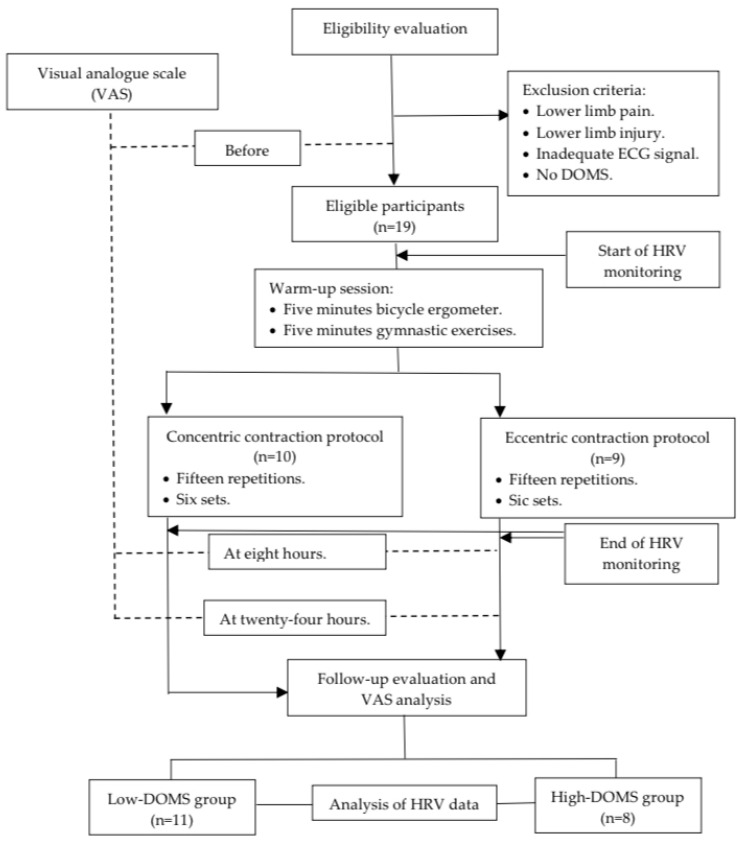
Flowchart of this study.

Before and after the data collection, the participants filled out a visual analogue scale (VAS) [37,38] questionnaire regarding the subjective determination of pain in the lower limb. Moreover, eight and twenty-four hours after the DOMS-inducing exercise protocol, the players were asked again about their pain based on the VAS scale (Figure 1). The VAS scales were evaluated as follows: 0—no pain, 1–3—mild pain, 4–6—medium pain, and 7–10—severe pain. The athletes were subsequently divided into two groups: those with DOMS values lower than 5 were assigned to the “low-DOMS” group, while those with values above 6 were assigned to the “high-DOMS” group (Figure 1). The data of the two groups were also analyzed separately in the current paper.

Prior to the dynamometer test measurements, a universal warmup protocol was conducted for every participant, including a 5-min bicycle ergometer task with minimal load and 5 min of basic gymnastic exercise performed without any additional weight (Figure 1). After the warm-up, measurements were executed with a Humac NORM isokinetic dynamometer (CSMi, Computer Sports Medicine, Inc., Stoughton, MA, USA) by controlling the movement of the knee in the dominant leg of the participants. All the participating athletes were familiar with the Humac NORM isokinetic dynamometer device; therefore, no prior training exercise was necessary. Ten participants executed a concentric contraction (CON) protocol and nine an eccentric (ECC) protocol. The measurement procedure in the isokinetic dynamometer was carried out as follows: 15 repetitions in 6 sets of CON or ECC while the motor moved the limb with an angular velocity of 60 deg/sec between a knee angle of 10 and 100 degrees (Figure 1). A minute rest interval was introduced between the sets. The participants were instructed to generate the maximal intensity of resistance while performing the ECC protocol and, similarly, the maximum intensity of push while the CON protocol was carried out.

### 2.3. Data Acquisition and Preprocessing

Electrocardiograms (ECGs) were recorded by MOX3 wearable ECG-Actigraph devices (Maastricht Instruments, Maastricht, the Netherlands), which non-invasively detected single-channel ECGs (sampled at 400 Hz frequency) and the participant’s motor activity. The dataset included ECG signals resampled to 2000 Hz using cubic spline interpolation to enhance R-peak detection accuracy [Accelerometry.eu. MOX3 Activity Monitor + ECG Logger. Available online: https://www.accelerometry.eu/products/wearable sensors/mox3/ (accessed on 21 May 2024)].

### 2.4. R-Peak Detection and RR Interval Calculation

R-peaks were identified using a custom detector, from which RR intervals and HR time series were derived. Artifactual beats and ectopic intervals were removed through a sequential segmentation procedure combined with the 30-point moving-median-based outlier rejection routine “isoutlier” in MATLAB R2020b version 9.9 (MathWorks, Natick, MA, USA).

### 2.5. Spectral and Complexity Analysis

Interpolated RR intervals, RR differences, and HR sequences were generated at 2 Hz using linear interpolation. Time–frequency analysis was performed using short-time Fourier transform (STFT) with overlapping windows (200-sample length, 10-sample step). Total power (TP), LF, and high-frequency (HF) spectral components were extracted for each window. Additionally, nonlinear dynamic complexity was quantified using SampEn. Entropy, in general, characterizes the unpredictability of the RR-interval time series (higher entropy = more complex signal), and among its various measures, SampEn is the most frequently used. More formally, SampEn(m, r, N) = ln(A/B), where A = N(d[RR_m+1_(i), RR_m+1_(j)] < r) and B = N(d[RR_m_(i),RR_m_(j)] < r). N is the number of vector pairs, while, in our case, the embedding dimension m = 2 and tolerance r = 0.2∙SD (standard deviation) [39]. Root mean square of successive differences (RMSSD) and standard deviation (SDNN) were also computed in each window.

### 2.6. Significance Analysis

In order to quantify the group-level differences between the calculated HRV parameters of the low-DOMS and high-DOMS groups, a 2D Kolmogorov–Smirnov test was applied. Subsequently, we calculated the mean HRV values of the two groups as a function of HR; then, the difference between the respective HRV(HR) curves representing the two groups was subtracted to visualize differences.

## 3. Results

### 3.1. The HR-Dependence Approach

**Figure 2:**
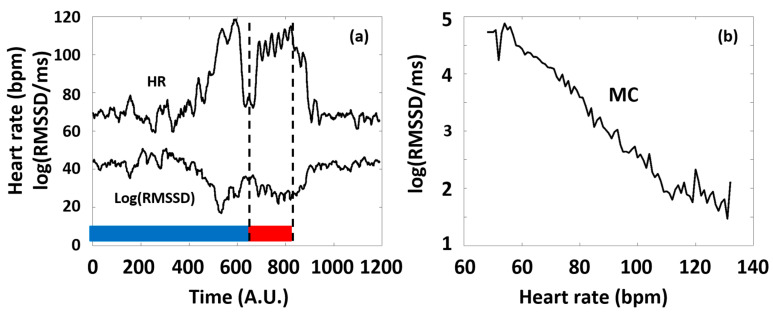
(**a**) Typical time series of HR and a corresponding HRV parameter (RMSSD) calculated from Holter ECG recordings of an individual athlete during the training session (blue and red curves, respectively). The 6 consecutive series of the targeted physical exercise bouts (Period B, red band) start around 650 s, followed by a relaxation period (after approximately 900 s). Data before approximately 650 s correspond to the preparation and the warm-up sessions (Period A, blue band). Note the complementary course of the two curves. (**b**) The RMSSD(HR) Master Curve (MC) was derived from the time series depicted in Figure 2a.

In our study, we considered typical time domain HRV parameters (RMSSD and SDNN), frequency domain parameters (LF and HF power), and a nonlinear parameter, SampEn, with special respect to the latter, as a function of HR. The primary reason for this is that all the HRV parameters are dependent on the actual HR at which they are measured, and this dependence is generally pivotal, namely, it usually masks the effects of all other factors on HRV [10,40,41]. Considering RMSSD as an example, Figure 2 illustrates our concept of depicting the HRV parameters of a selected typical individual as a function of HR (see Figure 2b), which considerably simplifies the representation of the otherwise complex dataset of HR(t) and HRV(t) functions (such as in Figure 2a), as well as the interpretation of the observed phenomena according to the ANS effects.

### 3.2. The Effect of Exercise on the HRV Parameters of the Athletes

**Figure 3:**
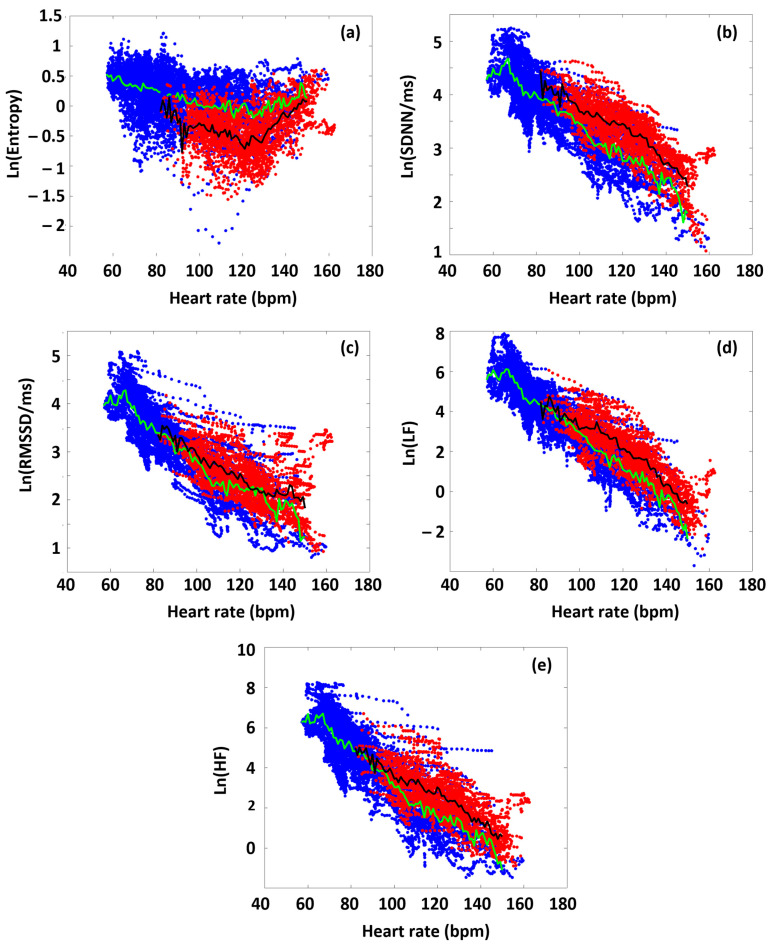
The HR dependence of the collated HRV data of the athletes on a semilogarithmic scale, before and during the targeted exercise session (Periods A and B, color-coded by blue and red, respectively. Green is the average of blue-coded data, while black is the average of red-coded data). (**a**) SampEn, (**b**) SDNN, (**c**) RMSSD, (**d**) LF, and (**e**) HF.

In the first approximation, we analyzed all the participants’ data to highlight differences in the various HRV parameters between time domains before and during the targeted exercise sections. Figure 3 shows the collected HRV data of all the athletes, as a function of HR, distinguishing the data obtained from the preparation/warm-up (Period A) and the targeted physical exercise (Period B) periods (blue and red dots, respectively). During Period A, SampEn decreased gradually as a function of HR (blue dots in the figure), up to a point around 120 bpm (turning point, HR_tp_), from where the entropy increased again. This type of entropy–HR relationship was observed for all athletes, but the HR range during the warm-up differed for each athlete, reflecting different parts of the entire curve for each person. It can also be seen that the SampEn value covers a wide range at a given HR. We also plotted the SampEn(HR) function during exercise (Period B), where the load also includes the rest between the six subsequent sets. During exercise, it can be observed that the entropy decreased compared to the state during Period A. In other words, increased exercise loading characteristically resulted in lower entropy at a given HR. This was true for the entire HR range examined, including the range below and above the turning point. Another interesting observation is that the entropy at a given HR was not only lower, but its value also remained within a much narrower range than in the case of no load (Period A) (Figure 3a).

The RMSSD, SDNN, LF, and HF parameters show a different pattern than SampEn, but they are very similar to each other, so we describe them together. The parameters measured during the warm-up (Period A) decreased linearly in a semilogarithmic representation as a function of HR, which confirms their exponential dependence [40]. This was also true for each athlete individually. However, under intensive exercise load (Period B), the values of each of these HRV parameters increased compared to the reference set in Period A. The increase in the time–frequency domain parameters, compared to the reference curve (Period A), due to exercise (Period B), suggests that ANS activity was increased under exercise over the entire examined HR range (the sympathetic–parasympathetic balance was upshifted compared to the normal state) [41].

### 3.3. DOMS-Related Differences in Typical HRV Parameters

As a next step, we performed an analysis to determine whether the degree of DOMS had an interrelationship with the HRV parameters determined before or during the exercise sessions. First, the HRV(HR) functions were determined for the preparation and warm-up period (Period A) from the RR time series data of both the low-DOMS and high-DOMS groups, separately; then, the natural logarithms of the respective group-averaged HRV(HR) curves were subtracted from each other (always the low-DOMS from the high-DOMS), after a proper normalization of the number of individuals in the two groups (Figure S1). Although the difference curves were noisy, a 2D Kolmogorov–Smirnov test made a clear distinction between the datasets of the two groups. In the case of all the parameters, H = 1, meaning that the two distributions are different (SampEn: *p* = 10^−19^, SDNN: *p* = 10^−26^, RMSSD: *p* = 10^−24^, LF: *p* = 10^−37^, HF: *p* = 40^−23^). A difference between the respective HRV(HR) curves representing the two groups could be visualized for all the ΔHRV parameters as a function of HR, including an apparent sign change around the turning-point HR value (HR_tp_).

**Figure 4:**
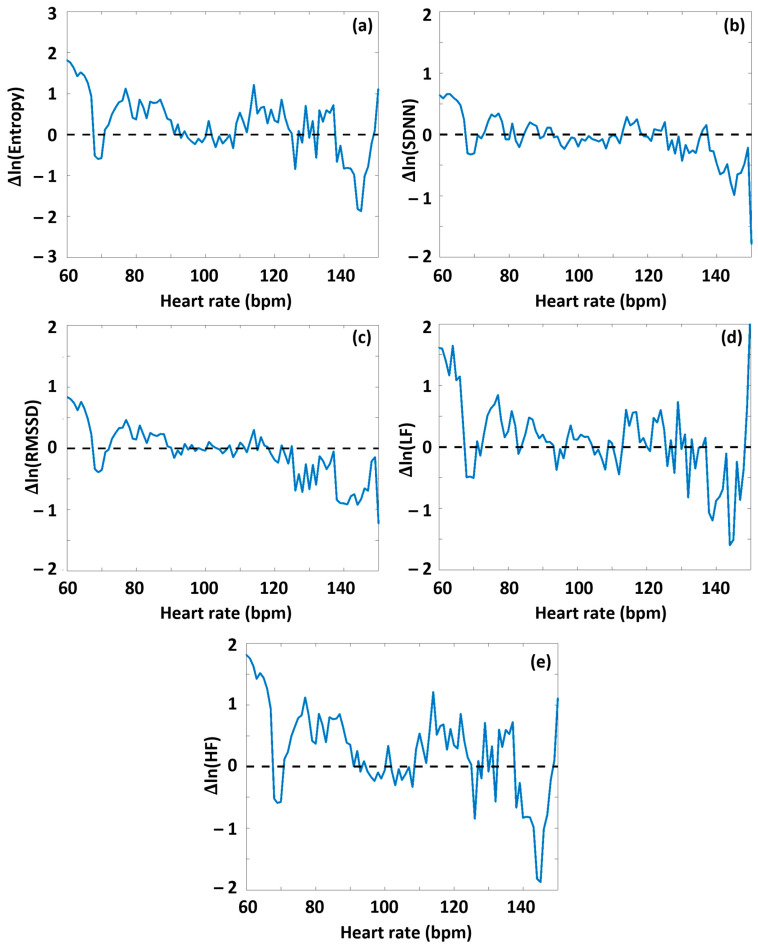
The effect of DOMS on various HRV parameters during the period of successive intensive exercise loading. The HRV(HR) functions were determined for Period B from the RR time series data of both the low-DOMS and high-DOMS groups, separately; then, the natural logarithms of the respective HRV(HR) curves were subtracted from each other, after a proper normalization of the number of individuals in the two groups. (**a**) SampEn, (**b**) SDNN, (**c**) RMSSD, (**d**) LF power, and (**e**) HF power.

Namely, during Period A, at HR values lower than HR_tp_, on average, greater values of all HRV parameters appeared for the high-DOMS group than for the low-DOMS group, while above the turning point, this relationship reversed. When performing the same type of evaluation on the data obtained during exercise (Period B) (Figures S2, S3 and Figure 4), a similar result arose for all the HRV parameters but entropy, where, interestingly, the difference curve describing the ΔSampEn(HR) function was practically mirrored to the abscissa. Namely, at HR values lower than HR_tp_, the averaged entropy values during exercise were somewhat higher for the low-DOMS group, while at values above HR_tp_, the entropy values of the high-DOMS group clearly dominated, increasingly with growing HR (Figure 4).

## 4. Discussion

The current study analyzed HRV measures during DOMS-inducing exercise. Our objective was to find alterations in these HRV parameters even during a DOMS-inducing exercise session despite pain evolving only post-exercise. We used an isokinetic dynamometer to induce DOMS in our research on 19 young male elite handball players who were continuously measured for HRV parameters during the applied exercise protocol. In order to establish DOMS-related differences in the HRV parameters of the athletes, we represented characteristic HRV parameters as a function of HR. While the HR dependence of the time–frequency domain parameters could be described by an exponential-like function, SampEn showed a V-shaped function, with a minimum (“turning point”, HR_tp_), separating descending and ascending intervals (before and after HR_tp_, respectively).

In the HR-dependent representation, it was apparent that intense exercise loading upshifted the time–frequency domain parameters of all the athletes in the entire HR range, contrary to the SampEn values, which were systematically downshifted, indicative of an upregulated sympathetic tone during the applied intensive exercise protocol. The standard deviation of the distribution of the group-level SampEn values was, in turn, found to be decreased, as well.

All the HRV parameters of the high-DOMS group showed significant group-level differences compared to those of the low-DOMS group, which, however, change sign around HR_tp_. The averaged SampEn(HR) function showed a nonlinear character under the DOMS-inducing exercise, with lower SampEn values for the high-DOMS group than for the low-DOMS group below HR_tp_, and vice versa above it. The HR functions of the corresponding time and frequency domain parameters showed an opposite tendency, i.e., turning from positive to negative around HR_tp_. Less prominent, but still significant, differences between the high- and low-DOMS groups could also be observed during the warm-up period.

Time domain parameters, such as RMSSD and SDNN, report on the size (amplitude) of HR fluctuations, while nonlinear parameters, such as entropy or the DFA exponent, inform us about the stochastic structure of HRV [10]. Frequency domain parameters, such as LF and HF power, on the other hand, form a link between the other two types of representation [10]. Nevertheless, whichever parameter one chooses to characterize the momentary HRV, it will strongly depend on the HR value, as demonstrated before [10,42,43,44,45]. There are a number of publications (e.g., [42,43,44,45]) discussing this problem, but they lack an exact mechanistic explanation that describes the relationship between HRV parameters and HR. Moreover, there has been no practical way to define a single “heart-rate-corrected” HRV parameter that could properly account for the HR dependence. Moreover, HRV–entropy reflects the orderliness of the “funny” current modulation; however, the underlying physiological processes that it depends on are not fully understood, and its HR-dependence has not been clarified either. The factors that can influence HRV–entropy can arise from ANS function, cellular processes (e.g., mitochondrial function, etc.), inflammatory factors during exercise, etc. Regarding the HR dependence of SampEn, some people associate the value of the turning point with the intrinsic frequency [45], which is also supported by the age dependence of entropy [10], although there is no unequivocal evidence for this claim. A decrease in entropy at a given HR under exercise may indicate altered ANS function and, especially, altered cellular processes. The current authors suggest the following mechanistic explanation for the abovementioned unexplained HR dependence: the ANS controls ultradian fluctuations through baroreflex sensitivity in an HR-dependent manner [32] and Piezo2, as an ultradian sensor (and Piezo1, as a diurnal sensor), exerts fine regulation over HR [33]. Not to mention, DOMS may occur with a transient proprioceptive neural switch, initiated on Piezo2 as the primary damage, including alterations in neurocellular metabolic and energy generation [24].

In an earlier study, signals were recorded for a several-hour-long period under natural, daily routine circumstances by a wearable ECG device (i.e., Holter monitoring, where otherwise no special measuring conditions were required), and data were used to derive a reference curve, called the “Master Curve” (MC) [40]. The MC, with the help of a nonlinear method based on modified Poincaré plots, was able to describe the HR dependence and appeared to be rather conservative for each individual from the daily to monthly scales [40]. In the earlier paper, it was suggested that the momentary deviations detectable on the minute scale from the MC could potentially be usable as a measure of changes in the dynamics of the heart rhythm, accompanying a disbalance of the ANS. Later on, this concept was applied to reveal stress- and relaxation-related physiological changes [41]. On the other hand, long-term dependence of the MC and other HRV parameters was described and interpreted as an age-related remodeling of the cardiac system [10], in line with the acquired Piezo2-channelopathy-induced quad-phasic non-contact injury model [9].

Earlier, it was proposed that LF power is not a reflection of sympathetic tone, as widely viewed even today, but rather the modulation of cardiac autonomic outflows by the baroreflex [46]. Moreover, in line with this proposition, it has also been suggested that LF power mostly reflects the excitatory activity level of Piezo2 in the baroreceptors and the heart [30]. Indeed, emerging research shows the presence and the functional role of Piezo2 in the heart [47,48]. Since a crosstalk is suspected between the proprioceptive system and the ANS through Piezo2 crosstalk [9,30], the novel unaccounted ultrafast proton-based, long-distance proprioceptive signaling in the nervous system [23] could be reflected in the cardiac autonomic outflows by the baroreflex in the form of proton release by Piezo2.

The LF power of HRV has been observed to dampen with age, as an indication of decreased ANS regulation of HR [46]; this also supports the aforementioned finding, leading to age-related remodeling of the cardiac system [10]. Moreover, it is known that under medium- to high-intensity exercise loading, LF power and SampEn take an inverse course. Accordingly, when LF power decreases almost to 0 value, SampEn is the greatest. This point is suggested to be the Piezo2 inactivation moment in order to maintain homeostasis and prevent pathological hyperexcitation [49]. It is interesting to note that Type Ia proprioceptive glutamatergic neurons are also GABAergic [50], and GABA may promote this inactivation on Type Ia proprioceptors, leading to the hyperexcitation of ASIC3-containing Type II proprioceptive fibers [9]. Under physiologic conditions, this increased sympathetic-loading-induced Piezo2 inactivation, reflected in the near 0 value of LF power, leads to triggered automaticity in SAN pacemaker cells, likely regulated by Ca_v_1.3 channels on a different pathway, and no longer by Piezo2 [49]. Furthermore, the Piezo2 inactivation at higher sympathetic loading intensities, induced by GABAergic inhibition [50], might reflect the desynchronization of the parasympathetic vagal and sympathetic regulation; hence, the cardiac sympathetic activation is left unopposed [49]. Moreover, the gradual inactivation of Piezo2 under this proposed ASR may trade fine motor movements for the “fight-or-flight” response [16,30]. In addition, the current authors suggest that this trade for the “fight-or-flight” response also sacrifices the Piezo2-dependent fine modulation of blood pressure and HR, or the abovementioned ultradian sensory regulation, through the baroreceptors.

In support of this, a recent traumatic brain injury (TBI) study revealed the essential contribution of PIEZO2 in the defensive arousal response (DAR) [51]. It is important to note that mild TBI is suggested to be an analogous bi-phasic non-contact injury mechanism, similar to DOMS, where the primary damage may also involve an acquired proprioceptive neuron terminal Piezo2 channelopathy [9]. This DAR mechanism is pivotal for survival, and it is activated by a perceived threat and evoked by visual and auditory cues in the presence of motor abilities [51]. This mechanism could be similar to the ASR implicated in DOMS [16]. A recent preprint paper emphasized the ultrafast matching of the Piezo2-initiated eye–brain, auditory/vestibular–brain, and proprioceptive muscle–brain axes within the hippocampal hub [13]. In addition, proprioceptive impairment, as a result of DOMS, is reflected in a tendency to mimic a positive Romberg test [13]. Correspondingly, with genetic manipulation, the aforementioned TBI study showed that reintroducing PIEZO2 promoted a reduction in escape latency and an increase in escape speed during DAR [51]. Indeed, neural Piezo2 was shown to activate DAR in association with enhanced motor abilities [51]. The current authors suggest that the TBI study may be supportive of Piezo2’s ultradian sensory and ultradian rhythm generation function and that Piezo2 channelopathy is why DOMS alters the response to postural perturbations [52] and significantly increases the medium latency response of the stretch reflex [53]. Moreover, the Piezo2-initiated heart–brain oscillatory axis has also been theorized [30,54]. The lack of integrating the principal ultradian backbone of this oscillatory heart–brain axis into the hippocampal hub is indicative of the finding that the degenerative heart failure condition with hippocampal damage is associated with short-term memory loss [55], reinforcing the role of the hippocampus in learning and memory. Here, we should add that Piezo2-induced repeated firing, in turn, causes local membrane tension changes and cytoskeletal rearrangements (actin dynamics), not to mention the osmotic shifts and swelling/shrinking of the neuron cell body. These mechanical and structural changes are sensed again by mechanosensitive ion channels, like Piezo2, in association with the pressure pulse detection capability of Piezo2 [56], completing the Piezo2-mediated feedback loop. Such positive-feedback loops serve as the bases of rhythm generation [57] and memory imprinting processes [58], as well.

It is worth considering that the aforementioned proton-release capability of Piezo2 is symmetry-breaking, causing the collapse of the disordered symmetric state in order to accomplish an ordered state; however, it is not symmetric, as acute intensive exercise loading increases. Moreover, Piezo2-released protons could initiate the novel unaccounted ultrafast proton-based, cross-frequency-coupled, long-distance proprioceptive signaling in the nervous system between proprioceptive Piezo2 and hippocampal Piezo2 at a distance, constructing the proprioceptive backbone of the muscle–brain axis [31]. As a distant analogy, reentrant superconductivity may arise in a superconductor and ferromagnetic layers at a higher (also lower) temperature phase, leading to paired oscillation in the space where the superconducting state presents a higher order compared to the normal state [59]. Similarly, this is why Piezo2 could be coined as a principal ultradian cross-frequency-coupler, or entrainer, under stress. However, the microdamaging event, leading to acquired Piezo2 channelopathy, breaks this function as the primary damage, resulting in proton reversal [9]. We suspect that this primary damage point may be reflected in HRV measures because the ultradian rhythm is likely transduced to the heart by the cross-frequency coupling of the muscle–brain axis, heart–brain axis, and the ANS through Piezo2–Piezo2 crosstalk in an HR-dependent manner. As another solid state physics analogy, the Piezo2 on proprioceptive terminals was coined to have a low-frequency Schottky semiconductor barrier diode-like function [23]. Indeed, super-Schottky diodes exist within superconducting tunnel junctions, not to mention in superconductor–semiconductor junctions [60]. Moreover, it is theorized that two-channel point-contact tunneling may exist in superconductors [61], further hinting at the possible schemes of the novel, unaccounted ultrafast proton-based, long-distance proprioceptive signaling in the nervous system [24].

Thereupon, the question arises as to what SampEn suggests in reference to this long-distance, ultrafast proton-based signaling mechanism. SampEn is an approximation of the complexity of time-series dynamic physiological signals [39]. The principality of Piezo2 in proprioception [25] is suggested to arise from its enigmatic feature, where only Piezo2 could initiate the aforementioned novel, unaccounted ultrafast proton-based, long-distance proprioceptive signaling within the nervous system [23]. The current authors suggest that this novel ultrafast proprioceptive signaling could be reflected in the cardiac autonomic outflows by the baroreflex, or LF power, in the form of proton release by Piezo2. Hence, SampEn may represent the Piezo2 modulating capability of excess entropy, and Piezo2 channelopathy, or proton reversal, impairs this modulating capability. By accounting for this, the current authors suggest that from the low- to moderate-load range, due to increasing Piezo2 activity, the orderliness of events increases monotonically with the extent of load, implying that entropy (measured as SampEn in HRV terms), in turn, decreases up to the point where Piezo2 channels are inactivated by the high strain of the membrane due to the increasing external load. From here on, the ordering action of Piezo2 channels ceases when the load further increases, and entropy starts to increase again, due to other physiological side effects (e.g., local heating or increased reactive oxygen species (ROS) production). We tentatively assign this turning point of the V-shaped SampEn(HR) curve to the inactivation of the Piezo2 channels. Note that below HR_tp_, LF power and SampEn are correlated, while above it, one finds an anti-correlation (see Figure 3 and Figure 4). It is noteworthy that the involvement of heat shock protein 70 (Hsp70) activation through the Hsp70/TLR4/Interleukin-6/TNF-α pathway is implicated in DOMS [62] and theorized as the route to Piezo2 channelopathy [15]. In addition, both the spontaneous activity-dependent interactions and the potentially life-threatening activity contribution, initiated by DRA or ASR, of ultradian rhythmicity increase the temperature of brown adipose tissue by approximately 1 °C [36]. Moreover, this elevation in temperature is associated with an approximately 0.8 °C increase in brain temperature and an 0.8 °C increase in body temperature [36], explaining the “heat of battle” response during DOMS-inducing exercise [16]. Accordingly, the low activity level of Piezo2, as the equivalent of high excitatory energy, keeps SampEn low on a reverse course, reflecting a peak in excess entropy. However, under acute intensive exercise loading, the excitatory activity level of Piezo2, as the equivalent of low excitatory energy, keeps SampEn on a high course, reflecting lower excess entropy.

Regarding the connection between the HRV parameters and DOMS, the first conclusion one may deduce is that below the turning point (HR_tp_), all the time–frequency domain parameters are greater for the high-DOMS group during the entire training, implying an increased ANS activity at low HR (Figure 3 and Figure 4). Above HR_tp_, however, an increasing level of autonomic attenuation develops as HR increases. This appears to correlate with the hypothesized increase in Piezo2 inactivation above HR_tp_. However, the entropy under intensive exercise load behaves the opposite way: the SampEn values of the low-DOMS group are somewhat higher at low HR values, but above HR_tp_, the SampEn level of the high-DOMS group starts to dominate greatly as HR rises. We suspect this reflects an increased level of Piezo2 channelopathy among the athletes in the high-DOMS group.

The delayed onset of pain mystery and the delayed involvement of nociceptive C-fibers in DOMS is an intriguing phenomenon regarding the link between pain and HRV alterations; therefore, understanding the underlying mechanism is important. Earlier, it was suggested that this delayed-pain sensation of DOMS and its associated delayed movement limitation, in the form of reduced joint range of motion and loss of force-generating capacity, as initiated by DAR/ASR, had evolutionarily provided 8 h of pain-free, limitless escape from danger in the wild [16]. The primary damage phase of the neurocentric DOMS mechanism theory may be initiated by a Piezo2 channelopathy on Type Ia proprioceptive terminals within the muscle spindle, leading to the secondary damage phase with harsher tissue damage in the extrafusal space, with the involvement of wide dynamic range (WDR) neurons on the spinal dorsal horn and other ion channels [9]. It is also important to note that an acquired Piezo2 channelopathy is likely the initiating peripheral input source that drives central sensitization on spinal nociceptive neurons, even in the case of DOMS [9,63,64]. Thereupon, acquired Piezo2 channelopathy is proposed to be the autonomous pain generator [9]. Indeed, the evolvement of pain and sensitization is lost as a consequence of loss-of-function mutations on PIEZO2 [65]. Furthermore, the aforementioned new neurocentric DOMS hypothesis heavily relied on the so-called gate control theory of pain [16], conceived by Melzack and Wall [66], and the activation of the aforementioned WDR neurons on the spinal dorsal horn [9]. Moreover, one finding of a research study showed a gate control mechanism of pain on the dorsal root ganglion (DRG) by proprioceptive neurons [50]. Interestingly, the study also theorized the presence of quantum tunneling in reference to the presented pain-gating mechanism [50]. However, the principal gate control of pain mechanism may reside further upstream on Type Ia proprioceptive terminals in the case of DOMS, where the acquired channelopathy of Piezo2 may lead to a neural switch and pain evolvement [9]. However, the pain evolves only as a result of the secondary damage phase of DOMS with nociceptive C-fiber contribution [9], when the aforementioned gate control mechanism of pain becomes critical downstream on the DRG, leading to WDR activation in the spinal cord [50]. This provides two pieces of evidence in support of the bi-phasic neurocentric DOMS theory. First, the presence of an intact intrafusal Type Ia proprioceptive terminal open gate cannot prevail for pain evolvement; hence, primary damage or Piezo2 channelopathy is a must. Second, extrafusal secondary damage is also essential for the activation of C-fibers in order for pain evolvement in DOMS [21].

An interesting translational pilot study investigated the sympathetic regulation of the DOMS effect [67]. One of the key findings was the evolved neurogenic inflammation with the involvement of the ANS, particularly the sympathetic nervous system (SNS) [67]. Correspondingly, the more the SNS was activated, the more the pain and inflammation were augmented [67]. Even more importantly, the applied stellate ganglion block (SGB) was capable of interfering with full DOMS evolvement [67]. In the current authors’ opinion, these results, based on a recently published paper [9], suggest that the acquired Piezo2 channelopathy on Type Ia proprioceptive terminal initiated the so-called inflammatory and even the gateway reflex during DOMS-inducing exercise. However, the pathway to the secondary damage phase was completely interrupted by the applied SGB, equivalent to irreversible Piezo2 channelopathy with no pain and sensitization [9,49]; hence, the so-called gateway reflex could not fully evolve. The data of our current research suggest increased SNS activity as a result of the DOMS-inducing exercise session and a correlation between SNS activity and pain (Figure 4 and Figure S1). We suggest that the increased SNS activity is likely driven by the unopposed Ca_v_1.3 ion channel due to the impaired Piezo2–Piezo2 crosstalk between the proprioceptive muscle–brain axis, heart–brain axis, and ANS.

We investigated the relevance of DAR and ASR as important underlying stress mechanisms. Essentially, personal differences in the stress response may exist, especially between trained and untrained individuals [68,69]. Since we followed a neurocentric tracing with regard to DOMS, the allostatic stress of neurons should be considered as well under prolonged sympathetic loading of eccentric (forced lengthening) contractions [9]. More specifically, two states of Piezo2 may prevail under allostatic stress, namely, inactivated intact Piezo2 and the acquired channelopathy of Piezo2 due to the prolonged eccentric nature of DOMS-inducing exercise [9]. The fundamentals of these two distinct states under allostatic stress were established by János (Hans) Selye by naming good stress as eustress and bad stress as distress [70]. Correspondingly, underlying Piezo2 channelopathy under allostatic stress is analogous to Selye’s bad stress, or the gateway to pathophysiology, leading to impaired Piezo crosstalk and the induction of the so-called gateway reflex [9]. On the contrary, inactivated Piezo2 under allostatic stress is analogous to Selye’s eustress that induces the so-called inflammatory reflex and leads to remodeling and adaptation within homeostasis [9]. The adaptive mechanism is performed by ASIC3 ion channels in terms of proprioception, as secondary proprioceptive ion channels [9,71]. It is noteworthy that previous research showed this secondary [71], protective role of ASIC3 in DOMS [72]. If we consider Piezo2 as a principal ultradian cross-frequency-coupler under stress [9], then good stress could be translated as frequency-coupled or fine-tuned stress buffering (intact underlying Piezo function and crosstalk, modulation primarily taken over by secondary ASIC3) in contrast to bad stress that is decoupled or disbalanced (impaired underlying Piezo2–Piezo1 and Piezo2–Piezo2 crosstalk, even when the modulation is taken over by secondary ASIC3). Piezo ion channels are evolutionarily conserved. Therefore, it is important to note that Piezo is the ion channel that buffers mechanical stress via modulation of intracellular calcium handling in the Drosophila heart, while the functional mutation of PIEZO fails to buffer mechanical stress, leading to pathological remodeling [73].

This demarcation line between good and bad stress is likely reflected in oxidative stress as well. Even the new neurocentric DOMS mechanism theory implicated the mitochondrial electron transport chain-generated free radical production as an important underlying factor in the evolvement of the primary damage [16], which was later coined Piezo2 channelopathy [21]. In fact, the loss of Piezo2 function impairs nitric oxide synthases and instigates remodeling [74], and this is suggested to be analogous to the acquired Piezo2 channelopathy, or the primary damage, of DOMS. Another important consideration is that ROS production in the mitochondria could induce high-frequency oscillations [75]. Therefore, this ROS-dependent mitochondrial oscillatory signaling transduction pathway should be accounted for in cardiomyocytes and myocytes under oxidative stress, like in the case of DOMS. Even more importantly, the impaired low-frequency Schottky semiconductor barrier diode-like function of Piezo2, as a result of Piezo2 channelopathy, may fail to modulate these ROS-dependent mitochondrial high-frequency oscillations. In support of this, it is evident that DOMS increases ROS production [76]. Moreover, the dual role of ROS in hippocampal learning and memory, more specifically, in long-term potentiation or the aforementioned memory imprinting, and hippocampal neurotoxicity, or even neurodegeneration, has been known for a long time, as well [77,78]. Not to mention, this dual role of ROS in the mitochondria of the heart is also present [79]. Importantly, the neurocentric DOMS theory emphasized that the primary damage could evolve at nerve terminals, like the Type Ia proprioceptive one, where the mitochondria content is high [16], leading to microscopically undetectable functional microdamage of Piezo2 [24]. The critical pathway of this hypothetical microdamage is electron leakage, serving ROS production essentially through the electron transport chain [16,23] and proton motive force [24], like in the case of the heart [79]. Piezo2 channelopathy is theorized to entail a proton affinity switch [24]; therefore, this can explain the dual role of ROS but also the loci of Piezo2 in neuron terminals packed with intracellular mitochondria that are indicative of the peripheral end of the abovementioned ultrafast axes. Acquired Piezo2 channelopathy was coined as the principal gateway to pathophysiology or the primary damage [9], as the common root cause of aging initiation [80]. Therefore, increased ROS production may contribute to the induction of the inflammatory reflex within homeostasis in support of remodeling; however, the proton affinity switch may induce the gateway reflex as a breach of remodeling [9]. Correspondingly, Piezo2 channelopathy may not only be the result of a proton affinity switch and the resultant neural switch, or miswiring, but it may also disrupt quantum tunneling of protons and electrons from mitochondria [24], leading to increased ROS production and the resultant increased entropy and accelerated aging. This later-mentioned pathophysiology is analogous to the one observed in the aging of brain mitochondria [81]. In addition, a proton affinity switch may be the reason why reverse electron transport may also prevail. Indeed, reverse electron transport increases mitochondrial ROS production [82].

A compelling finding showed that paired associative transcranial (TES) and peripheral electromagnetic stimulation (PES) diminished DOMS and facilitated the reinstitution of force generation [83]. On the contrary, PET alone without paired associative TES was not capable of providing any DOMS-related remedy [84]. Hence, the combination of TES and PES not only supports the neurocentric DOMS theory but also implies that the muscle–brain axis is also critical in the DOMS mechanism [71]. It has been presumed that sensing electromagnetic field-induced oscillating energy is conveyed by a common receptor, and, in return, this receptor could activate frequency-dependent biological pathways with the involvement of interfacial water [85]. Piezo2 was theorized to be this common receptor and the one that initiates ultrafast signalling in the nervous system [9]. In support of this theory, a recent study indeed showed that Piezo2 was the precise underlying mediator of magnetic stimulation [28]. The proton-based ultrafast signalling was also proposed to have a role in the force modulation of motoneurons [24]. Overall the paired associative TES and PES seems to reduce the secondary damage effect of DOMS, since the beneficial effect was detected within 72 h of DOMS induction [83], where the impairment of the Piezo2–Piezo2 crosstalk is implicated not only along the proprioceptive terminal–hippocampal axis [23] but between the intrafusal and extrafusal compartment as well [21]. Regarding the concrete biophysical mechanism of this non-synaptic, bi-compartmental Piezo2 cross-communication, we suggest the rapid proton-conducting Grotthuss mechanism as a potential candidate [86]. It practically refers to a concerted action of a large number of quasi-simultaneous proton hopping events along a H-bonded network of water molecules. The muscle spindles, with proprioceptive terminal Piezo2 content, are enclosed with a connective tissue capsule, which contains hygroscopic glycosaminoglycan (GAG) residues attached to collagen bundles [87]. These GAG residues, such as chondroitin sulfate, heparan sulfate, or hyaluronic acid side chains, are all of kosmotropic nature, according to Hofmeister terminology [88], forming a strongly H-bonded network of water molecules and giving rise to conduction of protons released by the Piezo2 channels. Moreover, the collagen bundles are themselves piezoelectric [89], i.e., they possess a large electric dipole moment along their axis upon external load, providing the asymmetry that will be able to define the directionality of proton conduction. In support of this, it is noteworthy that muscle spindles should be considered as a continuum with extrafusal space and not as an entirely isolated structure [21]. Indeed, intrafusal muscle fibers, for example, reach beyond the muscle spindle capsule into extrafusal territory and are tethered to the extrafusal connective tissue [90]. Extracellular matrix damage and impairment of the selective barrier of the muscle spindle capsule are likely part of the secondary damage phase of DOMS [9,21]. Therefore, combined TES and PES treatment seems to reinvigorate Piezo2 from its channelopathy and the resultant proton reversal by giving rise to proper proton conduction not only along the muscle–brain axis but also along the damaged bi-compartmental communication structures of Piezo2 ion channels. Finally, but not surprisingly, paired associative TES and PES treatment even improves HRV parameters substantially [26], likely by restituting the aforementioned Piezo2–Piezo2 crosstalk within the ANS.

## 5. Limitations

The aim of this study was to use the CON group as a control group initially. However, the 15 repetitions in 6 sets of the maximum-intensity CON protocol induced DOMS as well. Unfortunately, it turned out that six sets were too excessive for the CON group. This outcome is in agreement with earlier observations that at high exercise intensities, there is no difference in the change in muscle soreness from pre-exercise to post-exercise intervals between CON and ECC groups [91]. Since the sample size of the ECC would have been too low, the samples of the CON group were also included in this study.

Because of the unique approach of this study, no relevant prior research was available to conduct an appropriate power analysis. Therefore, to validate the adequate sample size with an appropriate power value (power > 0.8), we calculated a post hoc power analysis with the measured sample size using GPower. Only data for which a power value was greater than 0.8 were reported in this study. Additionally, the differences between the respective HRV(HR) point sets representing the low-DOMS and high-DOMS groups were quantified by a statistical method, namely, the 2D Kolmogorov–Smirnov test, and were found to be significant at the current sample size for all the HRV parameters used.

Moreover, the research group collaborated with the coaches of athletes and their athletic program. Therefore, some of the athletes were exposed to this study’s exercise protocol after days of training, while some of the athletes were exposed right after summer recess. This difference may have impacted the VAS score levels due to the repeated bout effect of DOMS with diminished symptoms [92]. However, this did not impact the mere fact of DOMS induction.

## 6. Conclusions

DOMS is a perplexing pain condition; still, a DOMS-specific alteration in HRV has not yet been detected during DOMS-inducing exercise activity in the scientific literature, in contrast to post-exercise HRV observations when DOMS fully evolves. We suggested, and then demonstrated, that HRV parameters, considered as a function of HR, can be used as sensitive measures of DOMS-related phenomena that occur under DOMS-inducing exercise and before the full development of pain. Consequently, the findings of the current study provide further indirect evidence for an initiating neural microdamage, also called primary damage, that prevails even under DOMS-inducing exercise. Furthermore, the diagnostic detection of this point may provide control over avoiding potential further injury risk in sport and exercise activities.

## Data Availability

The data that supports the findings of this study are available on reasonable request from the corresponding author. The data are not publicly available due to containing information that could compromise the privacy of underaged research participants.

## Supplementary Materials

The following supporting information can be downloaded at https://www.mdpi.com/article/10.3390/sports13080262/s1, Figure S1: The effect of DOMS on various HRV parameters during the preparation and warm-up period (Period A). The HRV(HR) functions were determined for period A from the RR time series data of both the low-DOMS and high-DOMS groups separately; then, the natural logarithms of the respective HRV(HR) curves were subtracted from each other, after proper normalization of the number of individuals in the two groups. (a) SampEn, (b) SDNN, (c) RMSSD, (d) LF power, and (e) HF power. Figure S2: The HR dependence of the collated HRV data of the low-DOMS athletes on a semilogarithmic scale during the targeted exercise session (Period B). (a) SampEn, (b) SDNN, (c) RMSSD, (d) LF power, and (e) HF power. Figure S3: The HR dependence of the collated HRV data of the high-DOMS athletes on a semilogarithmic scale, during the targeted exercise session (Period B). (a) SampEn, (b) SDNN, (c) RMSSD, (d) LF power, and (e) HF power.

## Abbreviations

The following abbreviations are used in this manuscript:

ANS	Autonomic nervous system
ASR	Acute stress response
CON	Concentric contraction
DAR	Defensive arousal response
DOMS	Delayed-onset muscle soreness
DRG	Dorsal root ganglion
ECC	Eccentric contraction
GAG	Glycosaminoglycan
HR	Heart rate
HRV	Heart rate variability
Hsp70	Heat shock protein 70
LF	Low frequency
MC	Master Curve
PES	Peripheral electromagnetic stimulation
REM	Rapid eye movement
ROS	Reactive oxygen species
SampEn	Sample entropy
SAN	Sinoatrial node
SGB	Stellate ganglion block
SNS	Sympathetic nervous system
TBI	Traumatic brain injury
TES	Transcranial electromagnetic stimulation
VAS	Visual analogue scale
WDR	Wide dynamic range
