# Supplementary material for: Heart Rate Variability Alterations During Delayed-Onset Muscle Soreness-Inducing Exercise—With Piezo2 Interpretation
Source: Sports (Basel). 2025 Aug 10;13(8):262. doi: 10.3390/sports13080262 (PMC12389744; doi:10.3390/sports13080262)
Supplement: Supplementary file 1 [file sports-13-00262-s001.zip › sports-3754431-supplementary.pdf]

Supplementary Information

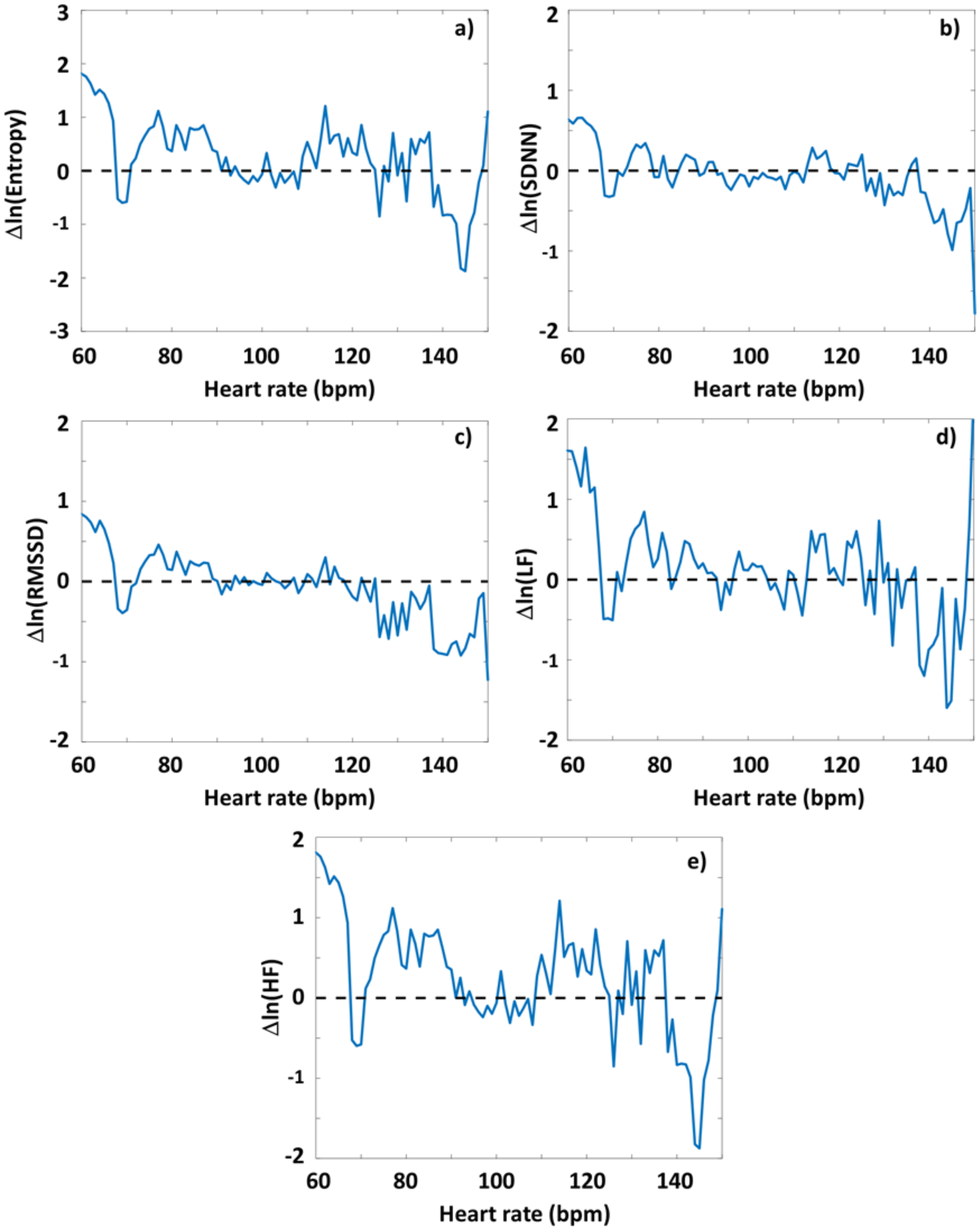

Figure S1. The effect of DOMS on various HRV parameters during the preparation and warm-up period (Period A). The HRV(HR) functions were determined for period A from the RR time series data of both the Low-DOMS and High-DOMS groups, separately, then the natural logarithms of the respective HRV(HR) curves were subtracted from each other, after a proper normalization to the number of individuals in the two groups. a) SampEn, b) SDNN, c) RMSSD, d) LF power, e) HF power.

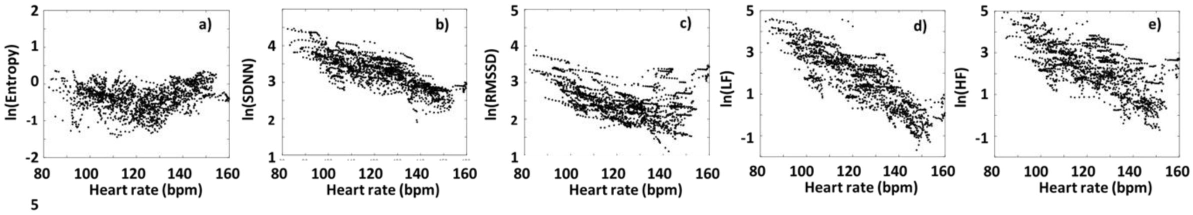

Figure S2. The HR-dependence of the collated HRV data of the Low-DOMS athletes on a semilogarithmic scale, during the targeted exercise session (Period B) a) SampEn, b) SDNN, c) RMSSD, d) LF power, e) HF power.

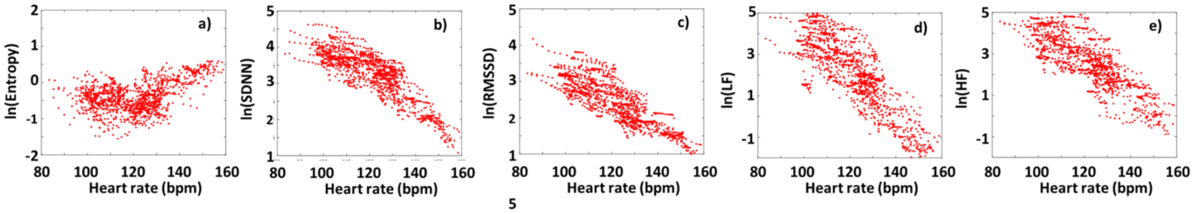

Figure S3. The HR-dependence of the collated HRV data of the High-DOMS athletes on a semilogarithmic scale, during the targeted exercise session (Period B) a) SampEn, b) SDNN, c) RMSSD, d) LF power, e) HF power.
